# Temporal stability of gel relaxation-time phantoms for quality control of T1 and extracellular volume fraction measurements

**DOI:** 10.1186/1532-429X-16-S1-P60

**Published:** 2014-01-16

**Authors:** Ee Ling Heng, Vassilis Vassiliou, Peter Gatehouse, Andreas Greiser, Shivraman Giri, Jackie Donovan, Sonya V Babu-Narayan, Michael A Gatzoulis, Dudley J Pennell, Sanjay K Prasad, David Firmin

**Affiliations:** 1 Cardiovascular BRU, Royal Brompton Hospital, London, UK; 2 CMR Unit, Royal Brompton Hospital, London, UK; 3 National Heart & Lung Institute, Imperial College London, London, UK; 4 Department of Adult Congenital Heart Disease, Royal Brompton Hospital, London, UK; 5 Siemens Medical Systems, Erlangen, Germany; 6 Siemens Healthcare, Chicago, Illinois, USA; 7 Department of Clinical Biochemistry, Royal Brompton Hospital, London, UK

## Background

Quantification of interstitial fibrosis by T1-mapping continues to gather momentum clinically with the recognition that extracellular volume fraction (ECV) measurements are pathologically elevated in a range of conditions. Well-known dependence of ECV estimation on imaging parameters requires quality-control by T1 phantoms[1-3]. These typically comprise agarose with NiCl[2] doping,[4] and are often used short-term, but with limited long-term data[5]. Such phantoms might assure long-term stability (over years) of methods applied in patients eg against scanner alterations. We therefore sought to assess their long-term T1 and T2 stability.

## Methods

**Figure 1:**
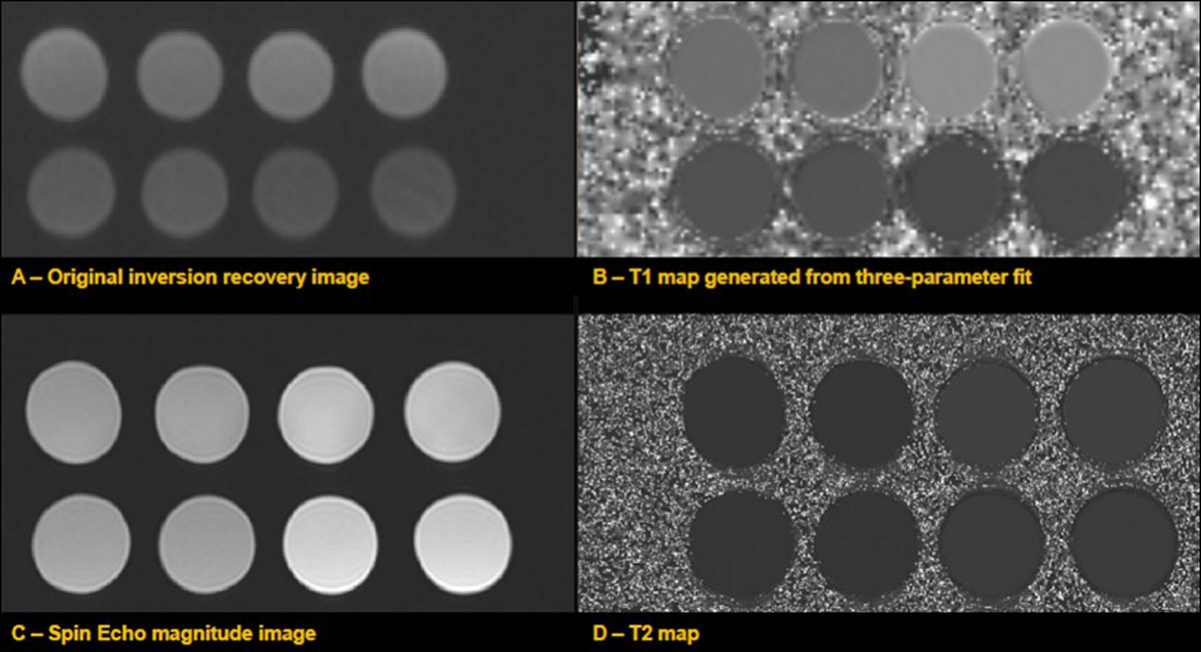
**T1 and T2 maps (B&D) generated from eight 11-cycle MOLLI images (A) and multiple TE spin-echo images (C)**. Two bottles of each tissue phantom were made because larger volumes of NiCl_2_-agarose gels improve composition accuracy. The bottles were averaged for T1 and T2 measurements.

NiCl_2_-agarose gel phantoms were made by a reproducible lab procedure, developed to model T1 and T2 of myocardium and blood, native and post-contrast[4,5]. Phantoms were 60 ml glass narrow-neck sealed thick-wall bottles filled without gaps from gel contraction while cooling. These were kept in the MRI room and imaged weekly 10 times (Siemens, Avanto 1.5T) using consistent coil and phantom arrangement, with 11-RR MOLLI: high-resolution (heart-rate 75 bpm) and low-resolution (100 bpm) versions, with pre-contrast and post-contrast variants,[6] plus spin-echo T2 *(*Figure 1*)*. Image parameters were identical weekly except automatic adjustments of flip-angle and reference frequency. Phantom mean T1 and T2 values were taken in pixelwise maps. The CoV (100 × Stdev/Mean)% of 10 weeks was compared against 10 re-positioned repeats. Temperature corrections[4] were estimated using MRI room recordings. Neither accuracy nor magnetisation transfer[7] were assessed in this study of long-term precision.

## Results

**Figure 2:**
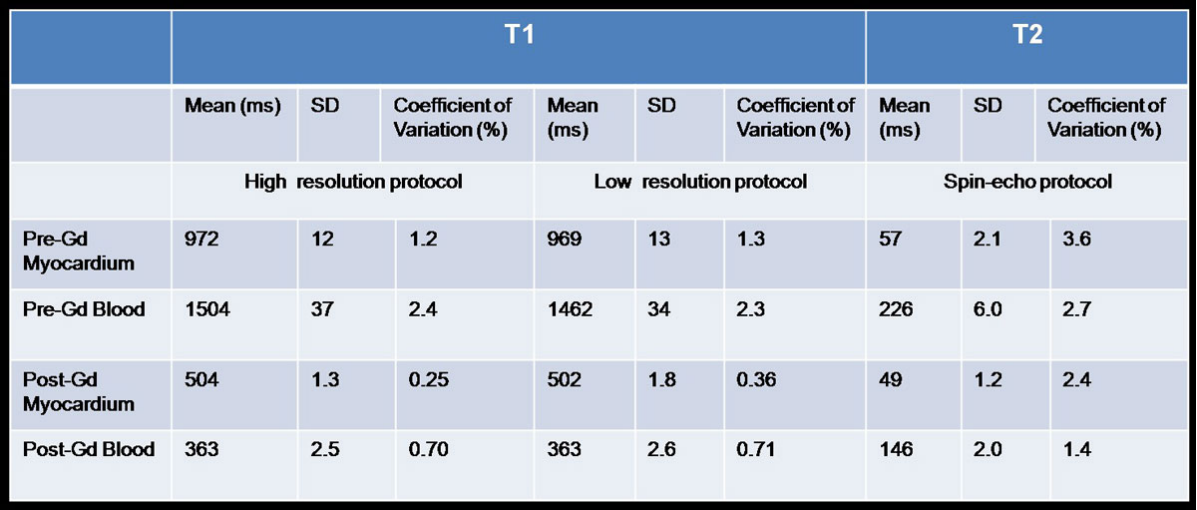
**The T1 values are from 11-cycle 8-image faster MOLLI protocols: preGad 5(3)3 and postGad 4(1)3(1)2**. These were repeated in high resolution (75 bpm) and low resolution (100 bpm) versions. The "overall CoV" stated in results is the average over all the CoVs(not all are shown). The "phantom ECV" was calculated using the averages of high and low-resolution T1s.

The T1 and T2 values *(*Figure 2*) *show greater variation over 10 weeks (T1 CoV 1.1% over all measurements) than short-term (T1 CoV 0.2%). The longer relaxation times showed more dependence on temperature. No consistent drift was observed over 10 weeks. For long-term usefulness, phantom scans would need to be more stable than the ≈1/10th change in native-T1 and ECV in many applications, and this was supported by the initial results. The 10-week native T1- phantom CoVs were ≈2%, while "phantom ECV" (Hct 0.43) predictably showed smaller CoV ≈1%. Multi-slice data showed minimal variation with location in the gels.

## Conclusions

Adequate stability of phantoms was shown over 10 weeks to support their continued use in ascertaining long-term precision of T1 mapping.

## Funding

NIHR Royal Brompton Cardiovascular Biomedical Research Unit and NHLI, Imperial College London.
